# Neurosyphilis and limbic encephalitis: practical guidance for diagnosis and treatment sequencing at an infection–autoimmunity interface

**DOI:** 10.3389/fneur.2026.1804075

**Published:** 2026-05-07

**Authors:** Alexis Demas

**Affiliations:** 1 Department of Neurology, Groupe Hospitalier du Havre, Le Havre, France; 2 ARGOS (Art, Research and Gestures Observed Scientifically) Neurosciences and Arts, Paris, France

**Keywords:** anti-NMDAR, autoimmune encephalitis, limbic encephalitis, neuroinfection, neurosyphilis, post-infectious autoimmunity, treatment sequencing, *Treponema pallidum*

## Abstract

Syphilis remains a re-emerging global infection with protean neurologic manifestations. Although neurosyphilis has long been framed as a direct infectious involvement of the central nervous system, an emerging case-based literature suggests that, in selected patients, *Treponema pallidum* infection may intersect with autoimmune limbic encephalitis, including anti–N-methyl-D-aspartate receptor (NMDAR) encephalitis. We conducted a structured narrative synthesis of published reports describing neurosyphilis presenting with a limbic encephalitis phenotype and reports documenting neuronal surface antibodies in the context of neurosyphilis. Across published reports, a recurrent phenotype is described: subacute psychiatric symptoms, memory impairment, seizures, mesial temporal abnormalities, and inflammatory cerebrospinal fluid profiles that do not reliably discriminate between infection and autoimmunity. While neurosyphilis can convincingly mimic autoimmune limbic encephalitis, a small number of observations describe limited improvement with antimicrobial therapy alone and more substantial recovery after immunomodulation, raising the possibility of an overlap phenotype in a minority of patients. Taken together, these case-based findings support a parallel diagnostic posture, securing antimicrobial coverage when neurosyphilis is supported, while preserving timely autoimmune phenotyping and treatment escalation when objective features suggest immune-driven persistence.

## Introduction

Syphilis, caused by *Treponema pallidum*, has regained epidemiological momentum despite effective antimicrobial therapy. After a marked decline in the mid-twentieth century, incidence has risen again over the last two decades, notably in high-income countries, reshaping the daily landscape of infectious neurology and reviving the clinical relevance of neurosyphilis as “the great imitator” (1–3). The neurological spectrum remains broad, and the diagnostic challenge is not confined to textbook presentations of meningovascular disease or general paresis. Rather, what complicates modern practice is the way neurosyphilis can borrow the phenomenology of other inflammatory syndromes, including those currently interpreted through an autoimmune lens (4, 5).

Autoimmune encephalitis is now a well-defined, treatable cause of subacute neuropsychiatric syndromes, supported by consensus diagnostic criteria that have sharpened clinical recognition and encouraged early immunotherapy when appropriate (6). The expanding recognition of neuronal surface antibody–mediated syndromes, most prominently anti-NMDAR encephalitis, has further reinforced the clinical reflex to consider autoimmune mechanisms when a “limbic signature” is present (7). In many centers, behavioral change, seizures, amnestic syndrome, and mesial temporal MRI involvement form a powerful attractor toward autoimmune encephalitis. Yet infections remain the crucial counterweight to this reflex, both because they can phenocopy autoimmune syndromes and because immunotherapy can be hazardous if infection is missed or incompletely treated (4–7).

Against this backdrop, a small but increasingly cited body of reports has described neurosyphilis presenting with a limbic encephalitis phenotype and, more provocatively, cases in which neurosyphilis coexists with neuronal surface antibodies, including anti-NMDAR antibodies (8–10). Other reports underscore that neurosyphilis can mimic autoimmune encephalitis with striking fidelity even in the absence of neuronal antibodies (11–13). These observations do not establish causality, but they disturb a comfortable binary. When limbic encephalitis unfolds in the setting of active or recent syphilis, are we always facing infection alone, exceptional mimicry, or sometimes infection plus autoimmunity, an overlap that changes diagnostic priorities and therapeutic timing? The present work addresses this bedside dilemma through a structured narrative synthesis and proposes practical guidance to reduce misclassification and avoid preventable disability.

## Methods

Because this phenomenon is uncommon and largely captured through case-based evidence, we performed a structured narrative review focused on publications describing neurosyphilis with a limbic encephalitis phenotype and publications reporting neuronal surface antibodies in the context of neurosyphilis. Searches targeted PubMed and reference lists of eligible articles, using combinations of terms including “neurosyphilis,” “limbic encephalitis,” “autoimmune encephalitis,” and antibody-specific terms such as “NMDAR” and “NMDA receptor.” We included adult reports and small series when the clinical presentation was compatible with limbic encephalitis, defined pragmatically as a subacute syndrome with psychiatric and/or cognitive symptoms and/or seizures supported by MRI and/or EEG context, and when the diagnosis of neurosyphilis was supported by clinical and laboratory elements as reported by the authors (4, 6).

For each eligible publication, we extracted narrative data on clinical phenotype, ancillary investigations (MRI, EEG, cerebrospinal fluid profile), immunological findings (neuronal surface antibodies in serum and/or CSF when performed), treatments (antimicrobial therapy and immunomodulation), and reported clinical evolution. Given the heterogeneity of reports and the aim of this work, which is to map a clinically consequential interface rather than estimate prevalence, synthesis was descriptive and interpretive, emphasizing recurrent patterns, diagnostic inflection points, and treatment-sequencing dilemmas.

## Results

### Neurosyphilis can inhabit the clinical silhouette of limbic encephalitis

The case literature suggests that neurosyphilis can occupy the clinical silhouette of limbic encephalitis. Patients are often described at the threshold where psychiatry and neurology merge, with subacute behavioral change, agitation or psychosis-like symptoms, seizures, and an amnestic syndrome that appears abruptly “too limbic to ignore” (11–13). Cerebrospinal fluid profiles frequently display inflammatory features that can be read as infectious but are also compatible with autoimmune encephalitis (6). EEG may reveal temporal slowing or epileptiform discharges, and MRI can show mesial temporal involvement, the radiological hallmark that in many clinical settings accelerates the autoimmune reflex (6, 11–13). In other words, neurosyphilis can convincingly reproduce the clinical and paraclinical grammar that modern neurology associates with autoimmune limbic encephalitis.

### The subset that drives the dilemma is defined by trajectory and immune signals

What distinguishes the subset of published cases that has driven renewed interest is not the presence of this grammar, since infection alone can account for it, but the trajectory under therapy and the immunological signals captured in some reports. A small number of observations describe clinical courses that were not fully explained by antimicrobial therapy alone, with improvement becoming more apparent after immunomodulatory treatment was introduced (8–10). Alongside these therapeutic narratives, a small number of reports document neuronal surface antibodies, most notably anti-NMDAR antibodies, detected in the setting of syphilis or neurosyphilis, raising the possibility that immune-mediated synaptic dysfunction can coexist with infection in selected patients (8, 9).

At the same time, the literature contains equally important counterpoints. Some cases received immunotherapy early because the presentation appeared autoimmune, only for neurosyphilis to later emerge as the unifying diagnosis and antimicrobial therapy to become decisive (11–13). These reports illustrate how easily neurosyphilis can be misfiled into autoimmune frameworks when syphilis testing is not reflexively included in early limbic encephalitis work-up.

## Discussion

### Three scenarios: mimic, overlap, and trigger

These observations support an infection–autoimmunity interface rather than a strict dichotomy (Table 1). The most conservative reading is mimicry, because neurosyphilis can reproduce the limbic phenotype with such fidelity that it can appear indistinguishable from autoimmune limbic encephalitis at first encounter (11–13). In this model, neuronal antibodies are absent, or when present at low titers are not necessarily causal, and patients improve primarily with antimicrobial therapy (4, 5).

**Table 1:** Mimic-overlap-trigger framework for neurosyphilis and limbic encephalitis.

Scenario	Core interpretation	Typical clues	Antibody testing posture	Treatment sequencing
Mimic	Infection-driven limbic phenotype	Improvement on adequate antimicrobials	Antibodies absent or low-titer/non-concordant	Prioritize antimicrobials; avoid pre-mature immunotherapy
Overlap	Infection plus immune-mediated overlap	Course disproportionate to adequate antimicrobial therapy	Convincing CSF neuronal antibodies with concordant phenotype	Treat infection promptly; add first-line immunotherapy after safety reassessment
Trigger	Post-infectious facilitation	Autoimmune phenotype emerging after infection	Antibodies may appear later; require serial testing	Treat infection; monitor; escalate immunotherapy if autoimmune syndrome consolidates

A second reading is increasingly difficult to dismiss, namely overlap. Selected reports describe neuronal surface antibodies, including anti-NMDAR antibodies, in the context of syphilis or neurosyphilis, and report improvement after immunomodulation, suggesting that in a minority of patients infection may coexist with an immune process contributing to symptom persistence or severity (8–10). This does not prove that syphilis causes autoimmune encephalitis, but it is sufficient to justify a clinical posture that keeps the overlap possibility live when objective features and trajectory remain discordant with adequate antimicrobial therapy.

A third hypothesis, more speculative but biologically coherent, is that neurosyphilis may act as a post-insult immune trigger in pre-disposed hosts, by analogy with the well-described sequence in which HSV-1 encephalitis can precede anti-NMDAR encephalitis, and more broadly with other CNS insults after which autoimmune encephalitis has been described (14, 15). This trigger model does not require asserting that *T. pallidum* directly “causes” autoimmune encephalitis. It requires accepting that a chronic inflammatory CNS infection may, under particular host conditions, perturb tolerance and facilitate immune activation toward neuronal targets.

### Mechanistic plausibility without overclaim

Mechanistically, chronic meningeal and perivascular inflammation can destabilize the blood–brain barrier and reshape antigen exposure (4, 5). Sustained innate immune activation may promote microglial reactivity and cytokine signaling that lowers thresholds for bystander activation and epitope spreading, while molecular mimicry remains a theoretical possibility. Host susceptibility may be decisive, because post-infectious autoimmune phenomena in neurology are typically subset effects, and immunogenetic associations have been reported in anti-NMDAR encephalitis (16). Although HLA typing is not systematically documented in published neurosyphilis-associated limbic cases, this lens offers a rational explanation for why overlap appears exceptional rather than ubiquitous.

### The HIV dimension: why the interface may be underestimated

Many patients with syphilis also live in epidemiological contexts where HIV coinfection is more frequent. This matters because HIV can broaden the infectious differential and make cerebrospinal fluid pleocytosis or protein elevation less specific for neurosyphilis or autoimmunity alone (5). In addition, immune reconstitution inflammatory syndrome (IRIS) after antiretroviral therapy initiation can itself generate new or worsening CNS inflammation and may mimic or coexist with an autoimmune trajectory, particularly when clinical deterioration appears paradoxical despite microbiologic treatment (17). HIV status should therefore not automatically preclude immunotherapy, but it should lower the threshold for renewed infectious reassessment, review of ART timing, and multidisciplinary discussion before escalation, especially in patients with advanced immunosuppression or recent immune reconstitution (17, 18).

### Antibody testing: where false certainty is most dangerous

The antibody signal is central to the overlap claim, but antibody testing must be interpreted with discipline. Autoimmune encephalitis criteria emphasize the need for clinical–paraclinical concordance, and they privilege CSF testing in syndromes like anti-NMDAR encephalitis because isolated low-titer serum positivity can be misleading in atypical contexts (6, 7). In neurosyphilis, where intrathecal inflammation and barrier dysfunction are common, it is especially important not to over-attribute pathogenic significance to weak, non-concordant antibody signals. Here, “convincing” CSF antibody positivity refers to detection of a neuronal surface antibody in CSF by a validated assay in a syndrome-concordant limbic phenotype, ideally with supportive serum–CSF concordance rather than isolated low-titer serum reactivity (6, 7). When this pattern is present and the trajectory remains disproportionate under adequate antimicrobial exposure, the probability of overlap rises and timely immune escalation becomes more defensible than prolonged diagnostic paralysis.

### Treatment sequencing: practical posture and protocol detail

**Figure 1:**
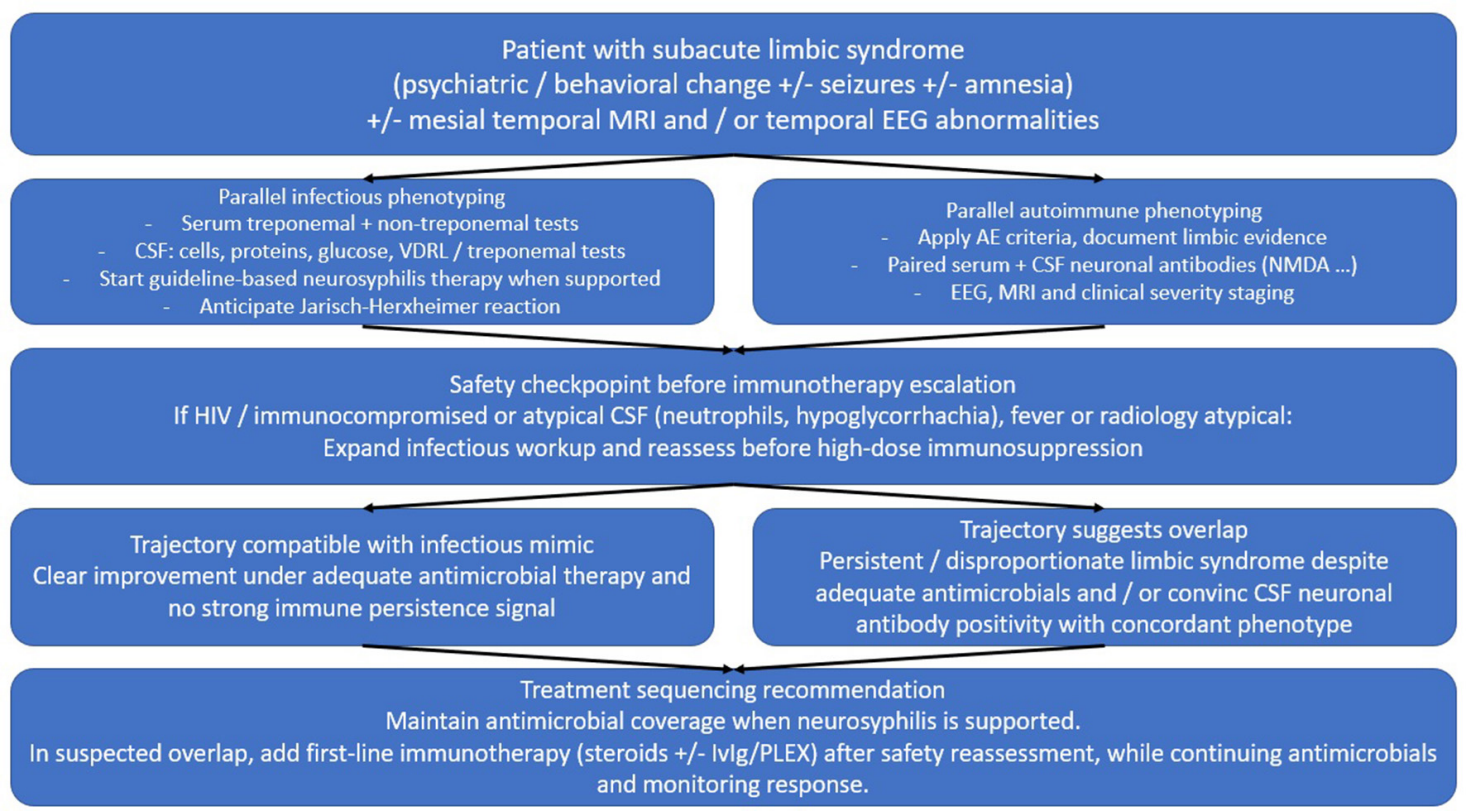
Proposed workflow for neurosyphilis-limbic encephalitis presentations. A parallel diagnostic and treatment-sequencing workflow for limbic encephalitis presentations in the context of active or recent syphilis. The workflow begins with immediate syphilis phenotyping and antimicrobial coverage when neurosyphilis is supported, while simultaneously assessing limbic involvement with MRI and EEG and performing paired serum and CSF neuronal antibody testing when the phenotype is strongly limbic or the course is disproportionate. The workflow includes reassessment after adequate antimicrobial exposure and specifies conditions that should trigger consideration of immunotherapy escalation in suspected overlap while requiring renewed infectious vigilance in immunocompromised hosts.

The clinical value of this synthesis lies in the posture it recommends. In a patient with a limbic encephalitis phenotype in the setting of active or recent syphilis, the question is not simply “infection or autoimmunity,” but “how do we evaluate both without losing time or causing harm.” This supports parallel phenotyping (Figure 1). Neurosyphilis evaluation and guideline-consistent antimicrobial therapy should proceed without delay when supported, while MRI, EEG, and neuronal surface antibody testing in serum and CSF should remain within reach when the phenotype is strongly limbic or when the clinical course appears disproportionate under appropriate antimicrobial coverage (4, 6).

On the infectious side, standard neurosyphilis therapy is penicillin-based intravenous treatment administered over an adequate duration, with alternative strategies discussed in state-of-the-art reviews when intravenous therapy is not feasible (4, 5). Clinicians should anticipate the Jarisch–Herxheimer reaction, because transient systemic and neurological worsening in the first day can confound early interpretation of “failure” if one expects immediate improvement. On the autoimmune side, first-line immunotherapy typically relies on high-dose corticosteroids and/or IVIG or plasma exchange depending on severity and local practice, and it should be considered when objective elements support immune-driven persistence rather than when the case merely “looks limbic” (6, 7).

A pragmatic sequencing approach is to secure antimicrobial coverage promptly, to proceed immediately with autoimmune phenotyping in parallel, and to reassess after adequate antimicrobial exposure. If the clinical course remains disproportionately severe or non-improving, if seizures and behavioral syndrome persist with limbic paraclinical signals, and if paired serum/CSF testing supports a neuronal surface antibody syndrome with clinical concordance, then immunotherapy can be escalated while continuing antimicrobial therapy and maintaining vigilance for missed infection, particularly in immunocompromised hosts.

### Data limitations and why case-based evidence remains clinically useful

The reviewer's concern about the level of evidence is correct. The literature remains largely case-based, and robust cohort studies quantifying prevalence or proving causality are not yet available. This limitation should be stated explicitly, not disguised, because it shapes what the paper can claim. At the same time, the absence of cohorts may reflect under-recognition and inconsistent reflex testing. If syphilis testing is not routine in early limbic encephalitis workups, cases will be misclassified as autoimmune encephalitis. If neuronal antibody testing is not considered in neurosyphilis cases with prominent psychiatric features and limbic imaging, overlap cases will be missed. A pragmatic framework can therefore have a dual function. It can reduce immediate bedside risk, and it can standardize recognition so that future cohorts become feasible and interpretable.

## Conclusion

Neurosyphilis can present as limbic encephalitis and can mimic autoimmune encephalitis with striking clinical and paraclinical fidelity in published case reports. The emerging literature also suggests that a minority of patients may fall into an overlap zone in which immune mechanisms plausibly contribute to the trajectory. In practice, a parallel diagnostic stance, treating neurosyphilis promptly when supported while preserving timely autoimmune phenotyping and escalation to immunotherapy when objective features suggest immune-driven persistence, offers a rational way to reduce misclassification and prevent avoidable morbidity.

## Data Availability

The original contributions presented in the study are included in the article/supplementary material, further inquiries can be directed to the corresponding author.
